# Eye Contact Affects Object Representation in 9-Month-Old Infants

**DOI:** 10.1371/journal.pone.0165145

**Published:** 2016-10-24

**Authors:** Yuko Okumura, Tessei Kobayashi, Shoji Itakura

**Affiliations:** 1 NTT Communication Science Laboratories, Nippon Telegraph and Telephone Corporation, Kyoto, Japan; 2 Department of Psychology, Graduate School of Letters, Kyoto University, Kyoto, Japan; University of Melbourne, AUSTRALIA

## Abstract

Social cues in interaction with others enable infants to extract useful information from their environment. Although previous research has shown that infants process and retain different information about an object depending on the presence of social cues, the effect of eye contact as an isolated independent variable has not been investigated. The present study investigated how eye contact affects infants’ object processing. Nine-month-olds engaged in two types of social interactions with an experimenter. When the experimenter showed an object without eye contact, the infants processed and remembered both the object’s location and its identity. In contrast, when the experimenter showed the object while making eye contact with the infant, the infant preferentially processed object’s identity but not its location. Such effects might assist infants to selectively attend to useful information. Our findings revealed that 9-month-olds’ object representations are modulated in accordance with the context, thus elucidating the function of eye contact for infants’ object representation.

## Introduction

Infants are sensitive to social cues, like eye contact, and pay close attention to these cues [1, 2]. Such social cues indicate someone’s communicative intention and play a primary role in facilitating social learning in young infants [3–6]. In addition, social cues can shape an infant’s likelihood of learning about events [7, 8].

Joint attention is one of the most critical contexts during parent-child daily interactions. After 9 months, infants often make eye contact with their parents and attend to their parent’s attention. Such joint attention provides the basis for the shared experiences necessary for many aspects of infant development and learning (e.g., [9, 10]). After establishing eye contact with others, infants can follow their gaze direction [5, 11–13]. Other studies reported that social engagement and joint attention with direct eye contact affect the efficient processing of object information by infants [5, 6, 14–18]. For example, Striano et al. [17] found that when an experimenter presented 9-month-old infants with an object by making eye contact and turning toward the object, infants looked longer at the familiar object than a novel object in the test phase, suggesting that joint attention with eye contact facilitated the infants’ object processing. However, little is known about what kinds of object information are influenced by joint attention.

In clarifying object processing, two neural routes for visual object processing must be considered: the object’s location and object’s identity (e.g., [19]). The dorsal route mainly processes the spatiotemporal properties of objects such as location, and the ventral route mainly processes the featural properties of objects such as identity [20]. In early infancy, infants are poor at integrating information processed separately in the dorsal and ventral routes [21]. Previous research has shown that spatiotemporal location has primacy over identifying features when infants process information about objects (e.g., [22–24]). Especially for infants under 1 year of age, since they might have difficulty integrating the two processes with object representations, they tend to rely on information about an object’s location rather than its identity [25, 26]. After 1 year of age, they can use featural information to individuate objects [27, 28], manifesting functional integration between object identity and object localization processing.

Recent research has shown an interesting finding that 9-month-old infants’ object representations are modulated by the context in which they perceive an object and retain different information about the object [29]. In their study using a violation-of-expectation paradigm, 9-month-olds were first shown short videos on a computer screen. In communicative trials, the experimenter greeted the infants by making eye contact and infant-directed vocalization and then approvingly pointed to an object. In non-communicative trials, the experimenter looked at the object and vocalized without looking at the infant and then unsuccessfully reached for the object. After a delay, an outcome was presented by revealing the same object in the same place (no-change outcome), the same object in a different location (location-change outcome), or a different object in the same location (identity-change outcome). The results demonstrated the differential reactions of the infants’ looking times at the outcomes in the communicative and non-communicative trials. That is, infants looked longer at the identity-change outcome than the no-change baseline in the communicative trial, whereas they looked longer at the location-change outcome than the no-change baseline in the non-communicative trial. These findings show that 9-month-olds detected the change of an object’s location rather than its identity in non-communicative situations, but in communicative situations they tended to focus more on its visual features than its location, suggesting a relative shift in object encoding in the two situations.

These results can be interpreted in the light of natural pedagogy theory [3, 30], which claims that infants learn general information applied to various situations (such as an object’s permanent, kind-relevant properties) rather than non-generalizable information in communicative contexts. An object’s identity is general and stable information when the object is recognized again. By contrast, since the current location of an object is variable, its spatiotemporal information is non-generalizable and irrelevant to its future recognition. Thus, in a communicative context, infants devoted their limited memory resources to encoding an object’s identity at the expense of encoding information about an object’s location. However, it remains unclear which of these communicative cues triggered the encoding shift from object locations to object identities, because multiple cues, including eye contact, infant-directed vocalization, and pointing gestures were used in the communicative context by Yoon et al. [29].

By focusing on the effect of eye contact as an isolated independent variable, the present study investigated how eye contact affects infants’ object representation. If eye contact influences infants’ object representation, such a demonstration would reveal the fundamental role of eye contact not only in social learning but also in object representations. In our task, 9-month-olds engaged in two types of social interactions with the experimenter, similar to the procedure described by Striano et al. [17]. In the eye contact condition, the experimenter made eye contact with the infant and looked at the object. In the no eye contact condition, the procedure was identical except that the experimenter looked over the infant’s head and then at the object, never making direct eye contact with the infant. We hypothesized that if eye contact alone substantially modulated the object representation, infants would selectively retain generic information about the object’s identity in the eye contact condition rather than in the no eye contact condition.

## Method

### Participants

The participants were 28 healthy, full-term Japanese 9-month-old infants (11 boys, 17 girls; mean age: 273.3 days; range: 258–291 days). Eight additional infants were tested but they were excluded from the analyses because of fussiness (n = 6) or procedural problems (n = 2). All the participants were recruited from a database of parents who agreed to participate in infant studies. Mothers were invited to participate in the experiment by phone and mail. All came from middle class families who lived in Japan’s Kansai region. We obtained written informed consent from the infants’ parents after explaining the content and the methods of the study. The study was approved by the Research Ethics Review Board at the Department of Psychology, Kyoto University.

### Apparatus and Stimuli

**Fig 1:**
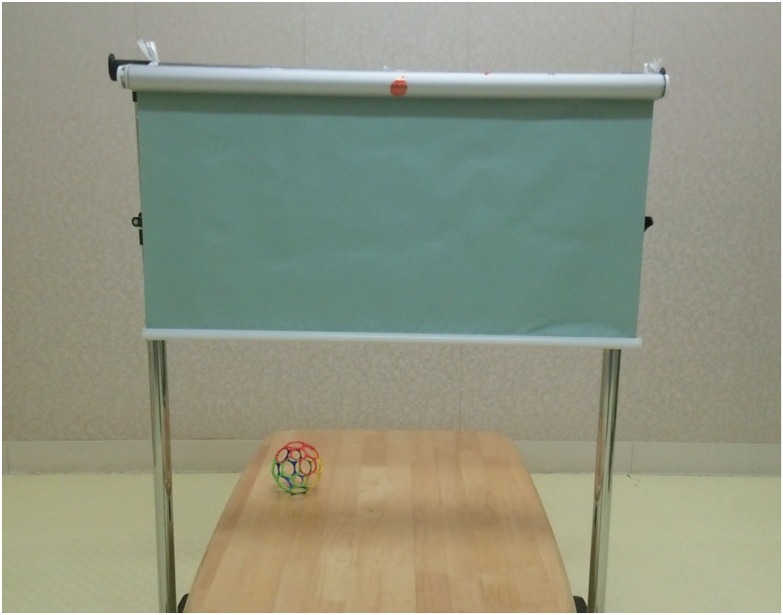
Experimental stimuli.

The infants were tested in a quiet room in an infant laboratory. A retractable screen was used to block the infant’s view while the experimenter placed and arranged objects on a table (Fig 1). The first video camera, which was located behind the experimenter, recorded the infant’s behavior, the object, and the experimenter. A second video camera, located behind the infant, recorded the experimenter’s behavior. The objects used in the study were stuffed animals or common plastic toys of similar size and shape (10 × 16 cm, 5.7° × 9.1° visual angle). To counteract any a priori preference for a particular object, the items that served as test objects were randomized across the infants.

### Procedure

Parents were instructed not to speak to or focus the infant’s attention to a particular spot in any way. In addition, they were naive to the experimental hypothesis. Infants were seated on their parent’s laps with an experimenter sitting directly opposite them across a table. The infants were given two practice trials and six experimental trials. During the practice trial, one object was placed midway between the infant and the experimenter in the center of the table. The experimenter raised the screen to reveal both herself and the object. She looked at the infant and the object and commented on it (e.g., “Look! I want to show you something interesting!” “There is a toy”). After 14 s, the experimenter lowered the screen to hide both herself and the object. The purpose of the practice trials was to familiarize the infants with the physical arrangements of the experimental setting, including the screen’s occlusion structure, and to expose the infants to the experimenter herself.

**Fig 2:**
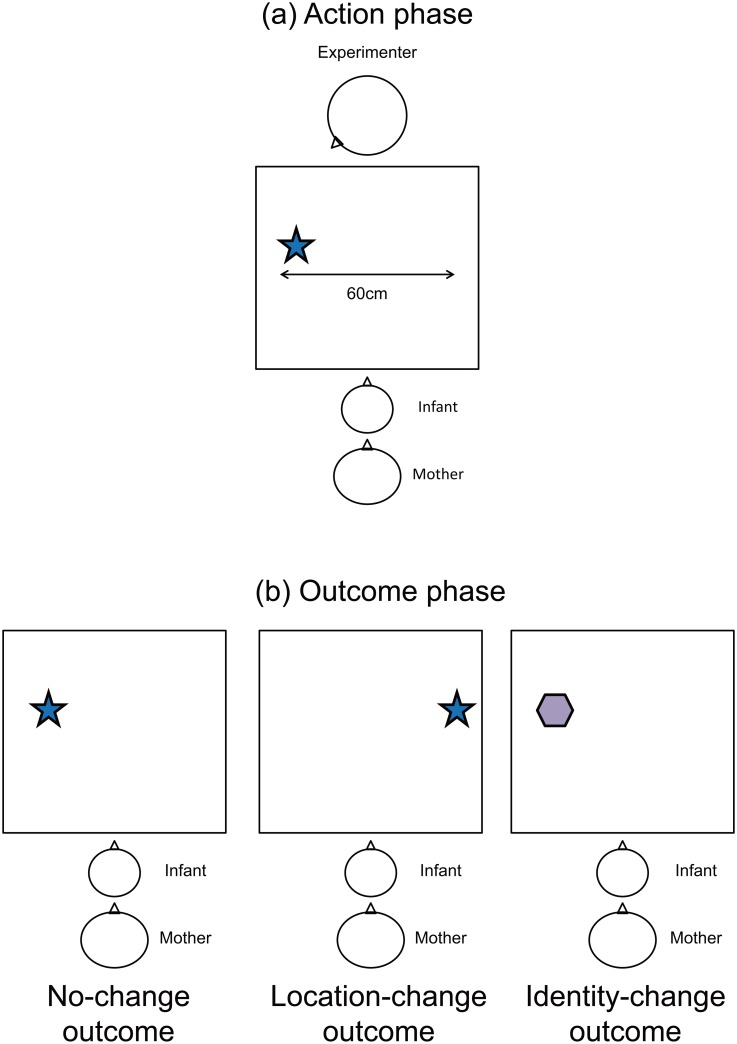
Experimental set-up for the action and outcome phases: (a) Experimenter made one of two object-directed actions (eye contact or no eye contact) in action phase. (b) One of three outcomes was presented in outcome phase, including no change in either location or identity (no-change outcome), a change in location but not identity (location-change outcome), or a change in identity but not location (identity-change outcome).

The experimental trial contained an action phase (for 14 s), a delay (for 5 s) during which both the experimenter and the object were hidden from view by a screen, and an outcome phase (for 15 s). See Fig 2 for the setup in the action and outcome phases. The action phase began when the screen was raised to reveal a single object and the experimenter (Fig 2a). The object was placed midway between the infant and the experimenter approximately 30 cm (17.1°) to the right or left of the table’s midline. The side on which the object was placed was counterbalanced across the participants. There were two types of action conditions. In the eye contact condition, the experimenter made eye contact with the infant (for about 2 s). She then turned toward and fixated on the object (for about 2 s), saying ‘‘There is a toy.” In the no eye contact condition, the experimenter looked about 30 cm above the infant’s head (for about 2 s). She then turned toward and fixated on the object (for about 2 s), saying ‘‘There is a toy” without making eye contact with the infant. The action phase lasted for 14 s, including three turning behaviors by the experimenter. The screen was then lowered to hide both the object and the experimenter.

After a delay of about 5 s, the outcome phase was conducted to assess the infants’ recollection of the hidden object. The screen was raised, revealing one of three outcomes (Fig 2b). The object that appeared was the same object in the same location as during the action phase (no-change outcome), the same object in a different location (location-change outcome), or a different object in the same location (identity-change outcome). After showing the object to the infant for 15 s, the screen was lowered and the outcome phase ended. The position (left or right) or identity of the objects was changed by the experimenter who was behind the screen and out of the infant’s sight. Since the object was placed approximately 30 cm to the right or left of the table’s midline in the action phase, its location in the location change outcome was moved 60 cm from left to right, or vice versa.

Overall, the infants participated in six trials that represented all the possible pairings of two action conditions (eye contact, no eye contact) and three outcomes (no-change, location-change, identity-change) in a within-subject design. The trial order was randomized across conditions and outcome types.

### Data analysis

Infants’ looking times were measured from the video recorded by the first camera that captured the infant’s face, the object, and the experimenter’s actions (backward). Their looking times toward each event phase were coded frame-by-frame at 30 frames per second for offline coding by two coders who were blind to the experimental conditions. In the action phase, infants’ looking times at the action event were coded until the phase ended (14 s). In the outcome phase, the infants’ looking times at the outcome event were coded until the infants looked away for 2 consecutive seconds or the phase ended (15 s). One coder scored the looking behaviors of all the participants, and the other independently scored a random 42% sample of the participants. The inter-rater reliability was 0.95 for gazing during the action phase and 0.91 for gazing during the outcome phase (Pearson’s correlation).

The difference between the eye contact and no eye contact conditions was whether the experimenter looked at the infants before turning toward the object. In the action phase, the experimenter’s turning behavior was repeated three times. If the experimenter could not make eye contact with the infants in the eye contact condition in all three times, the infants were excluded from the analysis. In addition, the infants who spent less than 30% of the time looking during the action phase were also excluded from the analysis.

## Results

### Action phase

**Fig 3:**
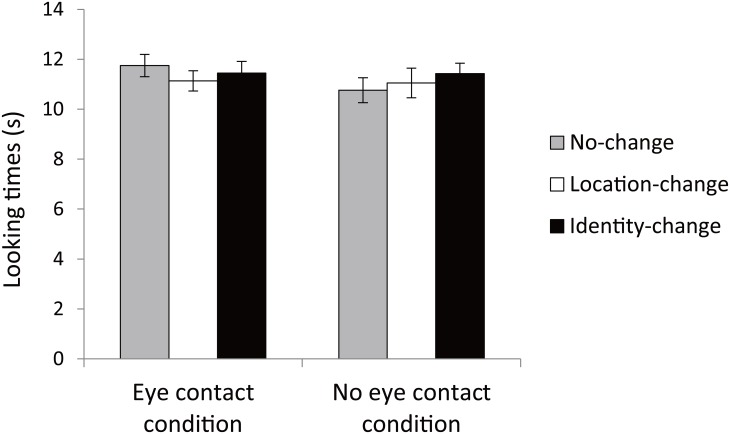
Mean looking times during action phase. Error bars represent standard errors.

The infants’ looking times during the action phase were entered into a 2 × 3 ANOVA with action conditions (eye contact, no eye contact) and outcomes (no-change, location-change, identity-change) as within-subject factors (Fig 3, see S1 Data). There were no main effects of action, *F*(1,27) = 0.92, *p* > .1, η_p_^2^ = 0.03, outcome, *F*(2,54) = 0.30, *p* > .1, η_p_^2^ = 0.01, or no interaction, *F*(2,54) = 1.14, *p >* .1, η_p_^2^ = 0.04. These results indicate that the infants attended equally to the action event regardless of the condition or the outcome.

### Outcome phase

**Fig 4:**
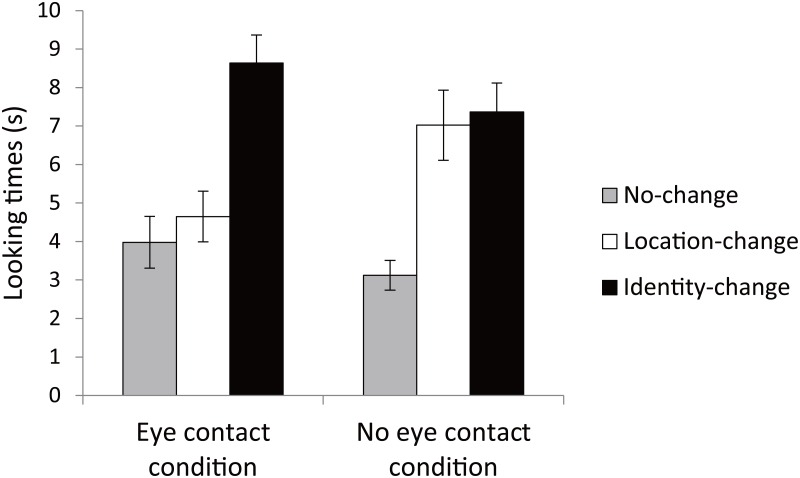
Mean looking times during outcome phase. Error bars represent standard errors.

We analyzed the infants’ looking times during the outcome phase by a 2 × 3 ANOVA with conditions (eye contact, no eye contact) and outcomes (no-change, location-change, identity-change) as within-subject factors (Fig 4). The results showed no significant main effect of condition, *F*(1,27) = 0.03, *p* > .1, η_p_^2^ = 0.001. In contrast, the results showed a significant effect of outcome, *F*(2,54) = 29.58, *p* < .01, η_p_^2^ = 0.52, and a significant interaction between condition and outcome, *F*(2,54) = 4.64, *p <* .05, η_p_^2^ = 0.15.

A one-way ANOVA revealed a significant outcome effect in the eye contact condition, *F*(2,54) = 16.82, *p* < .001, η_p_^2^ = 0.38. *Post hoc* analyses (Bonferroni) revealed that the infants’ looking times for the identity-change outcome were significantly longer than those for the no-change, *p* < .001, and location-change outcomes, *p* < .01, but their looking times for the location-change outcome did not differ significantly from those for the no-change outcome, *p* > .1, suggesting that infants in the eye contact condition selectively retained information about an object’s identity but not its location.

In the no eye contact condition, a one-way ANOVA also revealed a significant effect of the outcome, *F*(2,54) = 14.36, *p* < .001, η_p_^2^ = 0.35. *Post hoc* analyses (Bonferroni) revealed that the infants’ looking times for location-change and identity-change were significantly longer than those for the no-change outcome, *p*s < .001; there was no significant difference between the location- and identity-change outcomes, *p* > .1. These results suggest that infants remembered both the object’s location and its identity.

The above results were further confirmed by an individual level analysis. In the eye contact condition, 20 infants looked longer during the identity-change outcome than the other two outcomes, 6 infants looked longer during the location-change, and 2 infants looked longer during the no-change. The chi-square test revealed significant differences between outcomes, *χ*^2^ (2, *n* = 28) = 19.15, *p* < .01. *Post hoc* tests (Ryan) showed significant differences between identity-change and no-change, *p* < .001, and between identity-change and location-change, *p* < .05, but no significant differences between location-change and no-change, *p* > .05. In the no eye contact condition, 15 infants looked longer during the identity-change than the other two outcomes, and 13 infants looked longer during the location-change. The chi-square test revealed significant differences between outcomes, *χ*^2^ (2, *n* = 28) = 14.22, *p* < .01. *Post hoc* tests (Ryan) showed significant differences between identity-change and no-change, *p* < .001, and between location-change and no-change, *p* < .001, but no significant differences between identity-change and location-change, *p* > .05.

## Discussion

Our present findings demonstrate that 9-month-old infants retained different information about a novel object depending on whether their experience occurred in a social context with eye contact or in a similar context without eye contact. The infants detected a change in an object’s location and its identity when the object was shown in a context where the experimenter did not make eye contact with the infant. However, when the experimenter did make eye contact with the infant, they detected changes in an object’s identity but not its location. Thus, our findings suggest that 9-month-olds’ object representations were modulated based on the context in which they perceived an object. One might say that the infants in the eye contact condition did not detect the change of the object’s location because they did not look at the object in the action phase as long as in the no eye contact condition and did not have enough attention time to accurately process it. However, as supporting data, previous research has shown that infants follow the gaze direction of others toward an object more frequently when the other’s action is shown with eye contact compared to without it [5, 11]. Therefore, in the eye contact condition, infants are expected to look at the object, and they probably did have enough attention time to process it.

Our finding partly replicates the results of Yoon et al. [29], who showed a relative shift in object encoding depending on the contexts. However, because Yoon et al. [29] used multiple social cues in their communicative context, including pointing gestures, direct gaze, and infant-directed vocalization, it remains unclear which of these communicative cues triggered the encoding shift of the object representations. Our study focused on eye contact as an isolated independent variable and asked whether eye contact alone functioned as a social cue. We show that in a social context with eye contact, 9-month-old infants preferentially processed an object’s identity but not its location, clarifying that eye contact shaped the infants’ representations of novel objects. Regarding the object’s location and identity processing, previous research with infants under 1 year of age has shown that since they might have difficulty integrating the two processes with object representations, they tend to rely on information about an object’s location rather than its identity [25, 26]. Nevertheless, the 9-month-olds in our study processed object identity in a social context with eye contact. Therefore, we expect that eye contact can bias infants to process object identity.

A further issue concerns the underlying cognitive mechanisms by which eye contact shaped the representation of object identity. Our explanation is that eye contact induced infants to learn about the referents of generic information and shaped their object representations. This hypothesis is consistent with the theoretical framework of natural pedagogy [3, 30]. Indeed, infants’ sensitivity to eye contact in various contexts (e.g., [1, 11, 31]) implies that such social cues play important roles in initiating interactions and communicating information. Another explanation is that eye contact grabs infants’ attentional arousal and influences their representations without its interpretation in terms of forming communicative expectations [13]. Although the present study cannot discriminate between these possibilities, our results provide evidence that eye contact affects infants’ object representations.

Since an object’s identity generally belongs to its permanent properties, it is informative when recognizing the object again [3]. By contrast, since a moveable object’s current location is irrelevant for its future recognition, its location could be regarded as non-generalizable information. Eye contact has the power to induce the representation of object identity that is relevant for generalization at the expense of the representation of transient information about object location. Similarly, previous research has shown a special interpretation-modulating role for social cues in early social learning [32–34]. For example, infants at around 9-months persistently tend to search for an object in its initial hiding place even after observing the same object being hidden in another place (sometimes called the A-not-B error). Topál et al. [33, 34] concluded that social cues from the experimenter contribute to the emergence of such perseverative search error. Perseverative bias has been substantially reduced in the context without any social cues. These findings support the natural pedagogy idea [3], according to which perseverative error reflects a pragmatic misinterpretation of the experimenter’s hiding actions in social contexts as constituting a generalizable knowledge demonstration rather than just being a hide-and-search game.

The present study provides an interesting contrast to the infants’ looking time patterns in Yoon et al.’s study [29]. While Yoon et al. [29] reported that 9-month-olds detected the change of an object’s location rather than its identity in a non-communicative situation, the 9-month-olds in our study processed and remembered both its location and identity in situations without eye contact. Two possible reasons might explain these inconsistent findings. First, this difference might be related to the experimental settings. The participants in our study observed live demonstrations, while the infants in Yoon et al. [29] observed videos. Many studies have shown that infants’ ability to learn multi-step action sequences from a televised demonstration is much poorer than from a live demonstration [35, 36]; this is often called the *video deficit effect* [37]. Because our participants acquired adequate information from a live demonstration, they might have retained both an object’s location and its identity information. Second, in the non-communicative context, the experimenter in Yoon et al.’s study [29] performed reaching actions. As described in their paper, for such incomplete goal-directed actions as reaching, the location of goal-objects is considered more relevant than their identity because goal-directed reaches are often part of a larger plan intended to culminate in some action performed on the object by the actor herself. Thus, although 9-month-olds remembered both an object’s location and its identity, the reaching cue might preferentially bias them to retain locational information. Future research must examine the differences between live demonstrations and television/video presentations as well as the effect of reaching actions. Such attempts might elucidate the object-processing ability in infants, which is a fundamental cognitive capacity that forms the basis for complex thought and behavior. In addition, our future work must consider infants’ looking patterns. We measured the total looking times at the events, similar to past work [29], but the looking times at the entire scenes do not provide information about where and how infants are processing them. It would be interesting to explore where infants look in more detail depending on social contexts.

Taken together, our results show that direct eye contact markedly influences what infants learn from a situation. Direct eye contact cues biased infants to represent object identity that is relevant for generalization at the expense of transient object location. Notably, this does not mean that infants remembered more information in social contexts with eye contact, because they remembered both an object’s location and its identity features in a context without eye contact. The biases induced by eye contact might assist infants to process useful information preferentially from their environment. Our current findings clarify the function of eye contact in the acquisition of novel information.

## Supporting Information

S1 Data Individual data of looking times during action and outcome phases. (SAV)
